# Election of a Member in the Section of Operative Surgery

**Published:** 1846-12

**Authors:** 


*Election of a Member in the Section of Operative Surgery.*—M. Malgaigne was elected, after a spirited contest, which had attracted a full meeting. The result of the election was received with general satisfaction. The unsuccessful candidates, MM. Robert, Vidal de Cassis, and Manec, all distinguished surgeons, obtained a respectable number of votes.—*Lon. Med. Times.*

